# Clinical evaluation of early implant stability using resonance frequency analysis: a multicenter observational study

**DOI:** 10.3389/fdmed.2026.1812698

**Published:** 2026-08-05

**Authors:** Shobha J. Rodrigues, Vidya Kamalaksh Shenoy, Mithun K. Upadhya, Mihir R. Kulkarni, A. B. Tarun Kumar, A. A. Ponnanna, N. S. Mamatha

**Affiliations:** 1 Department of Prosthodontics, Manipal College of Dental Sciences Mangalore, Manipal Academy of Higher Education, Manipal, Karnataka, India; 2 Department of Prosthodontics, AJ Institute of Dental Sciences, Mangalore, Karnataka, India; 3 Department of Periodontology, SDM College of Dental Sciences and Hospital, Dharwad, Karnataka, India; 4 Bapuji Implant Center, Bapuji Dental College, Davangere, Karnataka, India; 5 Department of Prosthodontics, Krishnadevaraya College of Dental Sciences & Hospitals, Bangalore, Karnataka, India; 6 Department of Oral and Maxillofacial Surgery, Rajaraajeshwari Dental College and Hospital, Bangalore, India

**Keywords:** dental implant stability, ISQ, multi-center study, novel macro form, refirm implant, resonance frequency analysis, titanium grade 23

## Abstract

**Background:**

Implant stability is an important determinant of successful osseointegration and long-term clinical outcomes. Although novel implant designs continue to emerge, independent multicenter clinical evidence evaluating longitudinal implant stability patterns remains limited.

**Aim:**

To evaluate longitudinal implant stability patterns of a novel Grade 23 titanium dental implant system using resonance frequency analysis (RFA) across multiple clinical time points.

**Materials and methods:**

This multicenter prospective observational study included 230 patients receiving 295 implants across nine academic centers. Implant stability quotient (ISQ) values were recorded at implant placement, second-stage surgery, impression making, and prosthesis trial. Descriptive statistics and non-parametric analyses (Friedman, Wilcoxon signed-rank, and Mann–Whitney *U* tests) were performed. Spearman correlation analysis was used to evaluate associations between baseline and subsequent ISQ measurements.

**Results:**

ISQ values remained generally high throughout the observation period, with mean ISQ values ranging from 73.06 ± 11.66 at implant placement to 75.07 ± 8.23 at the prosthesis trial stage. Mandibular implants demonstrated higher ISQ values than maxillary implants during the early healing stages (*p* < 0.05), although these differences became less pronounced at later clinical stages. Positive correlations were observed between baseline ISQ values and ISQ measurements recorded at subsequent clinical stages. Thirteen implants failed during osseointegration and two after prosthetic loading, yielding a cumulative survival rate of 94.92%.

**Conclusion:**

Within the limitations of this multicenter observational study, the evaluated implant system demonstrated favorable longitudinal ISQ trends during early healing. However, long-term controlled clinical studies incorporating comparative implant systems, radiographic bone level assessment, and extended follow-up are required before definitive clinical recommendations can be established.

## Introduction

Dental implants are widely regarded as a predictable and effective treatment modality for the replacement of missing teeth, with long-term success dependent on the achievement and maintenance of osseointegration (1, 2). Implant stability, particularly during the early healing phase, is a critical determinant of treatment success and has been strongly associated with biological integration and long term clinical performance (3, 4).

Implant stability is influenced by several factors, including bone quality and quantity, implant macrogeometry, surface characteristics, implant dimensions, and surgical technique (5–7). Primary stability refers to the mechanical engagement between the implant and surrounding bone at the time of implant placement, whereas secondary stability develops through biological bone remodeling during healing. Recent clinical and experimental studies have further demonstrated that implant macro-design, diameter, and length may influence implant stability outcomes assessed longitudinally using resonance frequency analysis (8–10).

RFA is a non-invasive and reproducible method used to assess implant stability, and the resulting implant stability quotient (ISQ) values are commonly used as quantitative indicators of implant stability (11–13). In the present study, the term “stability pattern” refers to changes in ISQ values observed over sequential clinical time points. Contemporary investigations continue to support the use of RFA for longitudinal monitoring of implant stability during healing and prosthetic rehabilitation (14, 15).

Recent advances in implant design have focused on optimizing macro- and microstructural features to enhance mechanical engagement with surrounding bone. Design modifications such as tapered implant bodies, micro-threaded collars, internal conical connections, and surface treatments have been shown to improve primary stability, particularly in compromised bone conditions (16–19). However, much of the available evidence is derived from single-center studies or controlled experimental settings, limiting external validity and real-world generalizability (20). Consequently, independent multicenter clinical investigations are necessary to evaluate the reproducibility of implant stability outcomes across diverse clinical environments.

The Refirm dental implant system (Intessence, Bengaluru, India) is a recently introduced implant fabricated from Grade 23 titanium alloy, a material recognized for its favorable mechanical strength, fatigue resistance and corrosion resistance (21). The implant incorporates a hybrid tapered design, internal conical hex connection, platform switching, and a micro-threaded cervical region, all intended to optimize stress distribution and enhance early stability (22). Despite these proposed design advantages, independent multicenter clinical data evaluating longitudinal ISQ trends associated with this implant system remain limited. Therefore, the present multicenter prospective observational study aimed to evaluate longitudinal implant stability patterns assessed using resonance frequency analysis and ISQ measurements, at different clinical time points following placement of the Refirm dental implant system across multiple academic centers.

### Null hypothesis

There are no statistically significant differences in implant stability quotient (ISQ) values among the evaluated clinical time points, and there are no statistically significant differences in ISQ values between maxillary and mandibular implant sites.

## Materials and methods

### Study design and participant selection

This prospective, non-randomized multicenter observational was conducted in accordance with the ethical standards of the Declaration of Helsinki and STROBE reporting guidelines. The study was conducted across nine dental teaching institutions in India. Ethical approval was obtained independently at all participating centers. It was registered with the Clinical Trials Registry of India (CTRI/2021/08/035411).

### Clinician calibration and protocol standardization

All centers followed a standardized clinical protocol for patient selection, implant placement, resonance frequency analysis (RFA) measurements, and data collection. Investigators across all sites underwent calibration sessions to ensure consistency in surgical technique, implant stability measurements, and data recording methods. These calibration sessions included operator training regarding transducer placement, probe angulation, and recording of implant stability quotient (ISQ) values to minimize inter-operator variability. No site-specific deviations were reported.

### Participant selection

Participants requiring single-tooth replacement in mandibular and maxillary arch were recruited from from outpatient departments of the participating institutions. Written informed consent was obtained from all participants prior to enrollment.

Inclusion criteria:

- Adults aged ≥18 years
- Adequate bone volume (Lekholm and Zarb Class I–III)
- Healed extraction sites with a minimum healing period of 4 months prior to implant placement
- Presence of adjacent natural teeth
- Good oral hygiene, defined by absence of visible plaque accumulation and satisfactory compliance with oral hygiene instructions

Exclusion criteria:

- Uncontrolled systemic diseases, including uncontrolled diabetes mellitus
- Current or previous bisphosphonate or antiresorptive therapy
- Heavy smoking (>10 cigarettes/day) or substance abuse
- Active oral infections or untreated periodontitis
- Immediate implant placement
- Pregnancy or lactation
- History of head and neck radiotherapy

### Clinical protocol

A total of 295 implants were placed in 230 patients under standardized surgical protocols. Implant site preparation and placement were performed under aseptic conditions according to the manufacturer's recommended drilling sequence. All implants were placed at crestal bone level.

Only healed implant sites with adequate native bone volume were included in the study. Cases requiring simultaneous bone grafting procedures, guided bone regeneration, or sinus augmentation were excluded. Preoperative evaluation of available bone dimensions and bone quality was performed using cone-beam computed tomography (CBCT).

Postoperative medications, oral hygiene instructions, and follow-up protocols were standardized across all participating centers.

### Implant stability measurement

Implant stability was assessed using resonance frequency analysis (RFA) with the Penguin® RFA device (Integration Diagnostics AB, Sweden). A compatible MulTipeg™ transducer specific to the implant system was hand-tightened onto the implant according to the manufacturer's recommendations prior to each measurement.

ISQ measurements were recorded in both the buccolingual and mesiodistal directions at approximately perpendicular probe angulation relative to the transducer. To minimize measurement variability across centers, all operators underwent calibration and standardization training before study initiation.

Measurements were obtained at four clinical time points:

1. Implant placement
2. Second-stage surgery
3. Impression making
4. Prosthesis trial

The mean of the buccolingual and mesiodistal ISQ values was used for statistical analysis.

In the present study, initial ISQ values obtained at implant placement were considered indicators of primary implant stability.

#### Data collection and bone quality assessment

Sociodemographic details, medical history, habits, and soft tissue thickness (mean 2.39 ± 0.71 mm) were recorded. Bone density was assessed via CBCT and categorized accordingly. The distribution of the implant sites based on bone density is shown in Table 1.

**Table 1:** The distribution of the implant sites based on bone density.

S No	Bone density grade	Number of sites
1	D1	38
2	D2	154
3	D3	97
4	D4	6

### Statistical analysis

Statistical analysis was performed using SPSS software version 23.0 (IBM Corp., Armonk, NY, USA). Descriptive statistics were calculated for all variables. Since ISQ values did not demonstrate normal distribution, non-parametric statistical tests were applied. Continuous variables were expressed as median, interquartile range (IQR), mean, and standard deviation where appropriate.

Differences in ISQ values across sequential clinical time points were assessed using the Friedman test for repeated measures, followed by Wilcoxon signed-rank tests with Bonferroni adjustment for pairwise comparisons. Comparisons between maxillary and mandibular implant sites at individual time points were performed using the Mann–Whitney *U* test.

Spearman's rank correlation analysis was used to evaluate associations between initial ISQ values obtained at implant placement and ISQ values recorded at subsequent clinical stages. Correlation coefficients (*r*) and *p*-values were calculated.

A *p*-value ≤0.05 was considered statistically significant.

## Results

A total of 295 implants were placed in 230 patients, including 119 males and 111 females, with a mean age of 47.6 ± 12.3 years. Of the implants placed, 170 were located in the mandible and 125 in the maxilla.

Thirteen implants failed during the osseointegration phase and two implants failed after prosthetic loading, resulting in a cumulative survival rate of 94.92% at the prosthesis trial stage.

### Implant stability quotient (ISQ) trends

ISQ values remained generally high throughout the observation period, with mean ISQ values ranging from 73.06 ± 11.66 at implant placement to 75.07 ± 8.23 at the prosthesis trial stage. Although modest fluctuations in ISQ values were observed across clinical time points, the Friedman test demonstrated statistically significant differences between repeated measurements (*p* < 0.001) (Table 2). Spearman correlation analysis demonstrated positive associations between initial ISQ values obtained at implant placement and ISQ values recorded at subsequent clinical stages (Table 3).

**Table 2:** Implant stability quotient (ISQ) values across clinical time points.

Clinical time point	Mean ISQ	Standard deviation
Implant placement (Visit 2)	73.06	11.66
Second-stage surgery (Visit 4)	75.44	6.09
Impression making (Visit 5)	74.76	6.35
Prosthesis trial (Visit 7)	75.07	8.23

Values expressed as mean ± standard deviation. Friedman test demonstrated statistically significant differences across repeated measurements (*p* < 0.001).

**Table 3:** Correlation between baseline ISQ values and subsequent ISQ measurements.

Comparison	Spearman correlation coefficient (*r*)	*p*-value
Placement vs. second-stage surgery	0.62	<0.001
Placement vs. impression making	0.58	<0.001
Placement vs. prosthesis trial	0.55	<0.001

### Maxillary vs. mandibular implants

Mandibular implants demonstrated higher ISQ values than maxillary implants during the early healing stages, with statistically significant differences observed at implant placement and second-stage evaluation (*p* < 0.05). However, these differences decreased at later clinical stages (Table 4).

**Table 4:** Comparison of ISQ values between maxillary and mandibular implants at different clinical time points.

Clinical time point	Jaw	Mean ISQ	Standard deviation	*p*-value
Implant placement	Maxilla	68.10	11.57	<0.05*
	Mandible	73.65	9.33	
Second-stage surgery	Maxilla	70.90	11.96	<0.05*
	Mandible	76.15	6.35	
Impression making	Maxilla	71.80	5.20	NS
	Mandible	75.16	6.17	
Prosthesis trial	Maxilla	74.33	8.51	NS
	Mandible	75.14	8.14	

NS, not statistically significant. Comparisons performed using Mann–Whitney *U* test.

* Statistically significant (*p* < 0.05).

### Implant failures and survival

Among the 15 implant failures observed, 13 occurred during the healing phase before prosthetic loading, while two failures occurred after prosthesis delivery. Failed implants were more frequently associated with lower baseline ISQ values and maxillary implant sites; however, no statistically significant associations were identified with patient age or gender. No evident center-specific clustering of failures was observed.

## Discussion

This multicenter prospective observational study evaluated longitudinal implant stability patterns of a novel Grade 23 titanium dental implant system using resonance frequency analysis (RFA) across diverse clinical settings. ISQ values remained generally high throughout the observation period, with modest fluctuations observed between clinical stages. Although statistically significant differences in ISQ values were identified across time points, the magnitude of these changes was relatively small. These findings are consistent with previous studies reporting relatively stable ISQ trends during early healing and prosthetic rehabilitation phases (3, 11, 14).

Primary implant stability is a mechanical phenomenon largely dependent on implant–bone contact at the time of placement, whereas secondary stability develops through biological bone remodeling and maturation during healing. In the present study, initial ISQ values obtained at implant placement demonstrated positive associations with ISQ values recorded at subsequent clinical stages. Similar longitudinal associations have been reported in previous RFA-based investigations, suggesting that implants demonstrating favorable baseline ISQ values may tend to maintain clinically acceptable stability during healing (12, 14). However, because primary stability was assessed using ISQ values alone, these findings should not be interpreted as evidence of a causal relationship between mechanical primary stability and subsequent biological stability.

Recent investigations have emphasized the influence of implant macro-design and dimensions on implant stability outcomes. Quispe-López et al. demonstrated that implant length, diameter, and thread geometry significantly affect ISQ values assessed longitudinally using RFA (8). Likewise, Khan et al. reported that larger implant dimensions were associated with higher ISQ values at placement and during follow-up (9). The relatively favorable baseline ISQ values observed in the present study may therefore be partially related to the hybrid tapered macro-design, optimized thread configuration, and internal conical connection of the evaluated implant system, which are intended to enhance mechanical anchorage and stress distribution.

RFA remains one of the most widely accepted non-invasive methods for longitudinal monitoring of implant stability. Recent cohort and clinical studies have confirmed its reliability and reproducibility across different clinical stages and measurement systems. Reynolds et al. demonstrated consistent ISQ trends over a 3-year follow-up period using both Osstell® and Periotest™ devices, while Dhahi and Bede reported comparable stability assessments when RFA was evaluated alongside alternative systems such as AnyCheck® (10, 14). These findings support the use of RFA as an appropriate and reliable tool for longitudinal stability assessment in multicenter clinical investigations, particularly when the same implant system is evaluated over time.

Mandibular implants demonstrated higher ISQ values than maxillary implants during the early healing stages, which is consistent with established biological principles and previous reports attributing higher primary stability to greater cortical bone thickness and density in the mandible. However, these differences decreased at later clinical stages, suggesting that site-related differences may become less pronounced during healing. Bone quality remains an important determinant of implant stability and should be considered when interpreting ISQ measurements and planning loading protocols.

Implant material composition may also influence early mechanical behavior. The investigated implant system is fabricated from Grade 23 titanium alloy, which is characterized by favorable mechanical strength and fatigue resistance compared with commercially pure titanium. While titanium alloy implants have demonstrated satisfactory mechanical performance in previous studies, their direct influence on ISQ values and long-term biological outcomes remains incompletely understood. The present study therefore provides preliminary multicenter clinical data regarding longitudinal ISQ trends associated with this implant system without implying material superiority.

Unlike controlled randomized clinical trials, this investigation reflects real-world clinical performance across multiple academic centers, thereby enhancing external validity and generalizability. Nevertheless, several limitations should be acknowledged. The absence of a comparator implant system restricts direct comparison with other commercially available implants. The observational study design does not permit causal inference regarding implant design characteristics and stability outcomes. Additionally, the statistical analysis did not account for possible clustering effects associated with multiple implants placed in some patients. The follow-up period was limited to the prosthesis trial stage; therefore, long-term outcomes such as marginal bone level changes, peri-implant soft tissue stability, prosthetic complications, and implant survival beyond early loading could not be evaluated. Although calibration procedures were performed across participating centers, some degree of inter-operator variability cannot be entirely excluded. Furthermore, patient-reported outcome measures and functional loading parameters were not assessed.

Although favorable ISQ trends were observed during healing, these findings should not be interpreted as evidence supporting immediate or early loading protocols without further validation. Future controlled clinical trials incorporating comparative implant systems, radiographic bone level assessment, patient-reported outcomes, and extended follow-up durations are necessary to establish definitive clinical recommendations.

## Conclusion

Within the limitations of this multicenter prospective observational study, the evaluated Grade 23 titanium implant system demonstrated generally favorable ISQ values across sequential clinical stages as assessed using resonance frequency analysis. Although statistically significant variations in ISQ values were observed over time, the magnitude of these changes was modest. Mandibular implants demonstrated higher ISQ values than maxillary implants during the early healing stages; however, these differences became less pronounced at later clinical stages. While favorable longitudinal stability trends were observed, these findings should not be interpreted as evidence supporting immediate or early loading protocols. Further controlled clinical studies incorporating comparative implant systems, radiographic bone level assessment, and extended follow-up periods are necessary before definitive clinical recommendations can be established.

## Data Availability

The original contributions presented in the study are included in the article/Supplementary Material, further inquiries can be directed to the corresponding author.
